# Purple Potato Extract Suppresses Hypoxia-Induced Metabolic Reprogramming and Inhibits HIF-1α Signaling in Caco-2 Cells

**DOI:** 10.3390/nu17132079

**Published:** 2025-06-23

**Authors:** Qiaorong Cui, Qi Sun, Alejandro Bravo Iniguez, Xinrui Li, Min Du, Mei-Jun Zhu

**Affiliations:** 1 School of Food Science, Washington State University, Pullman, WA 99164, USA; qiaorong.cui@wsu.edu (Q.C.); qi.sun6@wsu.edu (Q.S.); a.bravoiniguez@wsu.edu (A.B.I.); 2 Department of Animal Sciences, Washington State University, Pullman, WA 99164, USA; xinrui.li1@wsu.edu (X.L.); min.du@wsu.edu (M.D.)

**Keywords:** purple potato extract, hypoxia, glycolytic metabolism, HIF-1α signaling, Caco-2 cell

## Abstract

Background: The hypoxia-inducible factor 1α (HIF-1α) pathway plays a key role in promoting glycolysis and tumor progression under hypoxic conditions in cancer cells. Purple potato (PP) extract, which is a polyphenol-rich natural product, has previously been shown to enhance mitochondrial function and suppress tumor growth in several cancer models. We hypothesized that PP extract could counteract hypoxia-induced glycolysis by targeting the HIF-1α pathway. Methods: Human colonic epithelial Caco-2 cells were treated with PP extract under hypoxic conditions, and its effects on glycolysis, oxidative phosphorylation, and HIF-1α signaling were evaluated. Results: Under hypoxia PP extract suppressed glycolysis, as evidenced by reduced lactate production and lower phosphorylated pyruvate dehydrogenase levels. In parallel, genes associated with oxidative phosphorylation were upregulated by PP extract, suggesting a metabolic shift under hypoxia. Additionally, PP extract reduced the protein accumulation of HIF-1α and its transcriptional activator XBP1 induced by hypoxia. Correspondingly, the expression of several HIF-1α downstream target genes, including *Vegfa*, *Pdk1*, *Ldha*, *Hk1*, and *Glut1*, was markedly reduced. Functionally, PP extract inhibited cell proliferation, migration, and drug resistance under hypoxic stress, indicating a broader inhibitory effect on hypoxia-driven malignant phenotypes. Conclusion: These findings suggest that PP extract disrupts cancer cell adaptation to hypoxia and supports its potential as a dietary approach against hypoxia-driven colorectal cancer, through further preclinical studies are warranted.

## 1. Introduction

Colorectal cancer (CRC) poses a significant public health challenge because of its high incidence and death rate [1]. CRC progression occurs in multiple stages, with the initial phase often characterized by excessive epithelial cell proliferation [2]. During neoplasia, the development of the vascular system cannot keep up with the rapid proliferation of cells, resulting in an avascular environment characterized by insufficient oxygen and nutrient supply, ultimately causing hypoxia stress [3].

In response to hypoxia, neoplastic cells initiate adaptive mechanisms to survive, primarily through the hypoxia-inducible factors (HIFs) pathway [4]. HIFs are transcription factors that respond to cellular oxygen levels and regulate the expression of numerous genes involved in angiogenesis, glucose metabolism, erythropoiesis, and cell survival [4]. In mammals, three oxygen-sensitive HIF-α subunits have been identified: HIF-1α, HIF-2α, and HIF-3α, with HIF-1α being the most extensively studied and broadly implicated in cancer progression [5]. HIF-1α is rapidly degraded under normoxia via the ubiquitin–proteasome pathway but stabilized during hypoxia. Upon stabilization, HIF-1α translocates to the nucleus, dimerizes with HIF-1β, and activates the transcription of hypoxia-responsive genes that facilitate cellular adaptation to low-oxygen environments [6,7,8].

In colorectal cancer, the expression of HIF-1α has been shown to increase as the distance from blood vessels expands, creating an oxygen and nutrient gradient [9,10]. This hypoxic microenvironment not only drives metabolic reprogramming but also contributes to therapy resistance and cancer aggressiveness. Mechanically, hypoxia coupled with nutrient deprivation triggers the unfolded protein response (UPR) and endoplasmic reticulum (ER) stress [11]. The activated UPR enhances cancer cell survival under stress conditions and has been associated with reduced sensitivity to chemotherapeutic agents [12,13]. Specifically, the transcriptional activator X-box binding protein 1 (XBP1), activated by UPR-induced inositol-requiring transmembrane kinase/endoribonuclease 1α (IRE1α) [14], forms a transcriptional complex with HIF-1α. This complex drives the expression of HIF downstream target genes, such as vascular endothelial growth factor a (*Vegfa*), pyruvate dehydrogenase (*Pdh*), pyruvate dehydrogenase kinase 1 (*Pdk1*), hexokinase 1 (*Hk1*), lactate dehydrogenase A (*Ldha*) and glucose transporter 1 (*Glut1*) [15].

By activating the expression of those genes, HIF-1α promotes a metabolic shift toward glycolysis (also known as the Warburg effect), even in the presence of oxygen. This shift allows cancer cells to generate energy rapidly while avoiding mitochondrial oxidative metabolism, which is often compromised under stress [16]. Additionally, the increased glycolytic flux supports biomass synthesis and reduces reactive oxygen species production, further enhancing cancer cell survival and proliferation [17]. Beyond metabolic adaptation, HIF-1α activity enhances tumor cell migration, angiogenesis, immune evasion, and resistance to conventional therapies, including chemotherapy and radiation [16,17,18].

Targeting the HIF-1α pathway and its associated metabolic consequences has therefore emerged as a promising strategy for anticancer therapy. Natural products, particularly those rich in polyphenols, are gaining attention for their multi-targeted effects on cancer metabolism and signaling pathways [19,20]. Our previous study in Caco-2 cells demonstrates that polyphenol-rich purple potato (PP) extract activates the AMP-activated protein kinase/peroxisome proliferator-activated receptor-γ coactivator (PGC-1α) axis, promoting mitochondrial biogenesis and oxidative phosphorylation [21]. These findings highlight PP extract as a potential metabolic modulator. However, the impact of PP extract on cancer cell metabolism under hypoxic conditions—particularly its influence on HIF-1α signaling and glycolysis—remains unexplored. This study aimed to evaluate the effect of PP extract on glycolysis and HIF-1α signaling induced by hypoxia. We hypothesized that PP extract inhibits colon cancer cell migration, proliferation, and drug resistance under hypoxia by suppressing glycolysis and HIF-1α signaling.

## 2. Materials and Methods

### 2.1. Preparation of Purple Potato Extract

The PP extract was prepared following the previously described method [21]. Briefly, raw Purple Pelisse purple potatoes, grown in the Pacific Northwest and typically harvested in September, were diced (with skin), freeze-dried, and grounded into powder, defatted, and extracted using 80% ethanol containing 1% formic acid (Sigma; St. Louis, MO, USA). The total polyphenol content in the resulting PP extract was quantified using the Folin–Ciocalteu assay, and the concentration was expressed as μg/mL, with chlorogenic acid equivalent [22]. As reported in our previous study, the total polyphenol content of the PP extract was 14.75 ± 0.30 mg chlorogenic acid equivalents (CGAeq) per gram of dry weight of lyophilized purple potatoes [22].

### 2.2. Cell Culture

The human colonic epithelial cell line Caco-2 was obtained from the American Type Culture Collection (Manassas, VA, USA) and routinely maintained in Dulbecco’s Modified Eagle’s medium (DMEM, Sigma) supplemented with 10% fetal bovine serum (FBS, Sigma), 100 units/mL penicillin G, and 100 μg/mL streptomycin (Sigma) at 37 °C in a 5% CO_2_ atmosphere.

For treatments, Caco-2 cells were seeded at 1 × 10^6^ cells per well in 12-well plates and allowed to grow in complete DMEM for 12 h prior to treatments. Cells were treated with 40 μg/mL PP extract or left untreated as a control. For hypoxia treatment, cells were cultured in a hypoxia chamber equipped with a flow meter (STEMCELLTM Technologies Inc., Seattle, WA, USA). The chamber was flushed with a tri-gas mixture containing 1% O_2_, 5% CO_2_, and 94% N2 (A-L Compressed Gases, Boise, ID, USA) for 4 min at a flow rate of 20 L/min. Cells were maintained in the chamber at 37 °C. For normoxic conditions, cells were incubated at 37 °C with 5% CO_2_ and 20% O_2_ using a standard cell culture incubator. Cells were collected 12 h after treatments for immunoblotting and quantitative reverse transcription PCR (qRT-PCR) analysis, and 48 h post-treatment for extracellular metabolite analysis.

### 2.3. Immunoblotting

Immunoblotting analysis was performed as previously described [22]. Primary antibodies against pyruvate dehydrogenase (PDH, #2784), phosphorylated PDH (p-PDH, #31866), IRE1α (#3294), and spliced XBP1 (XBP-1s, #40435) were obtained from Cell Signaling Technology (Beverly, MA, USA). The HIF-1α antibody (#PA1-16601) was obtained from ThermoFisher Scientific (Waltham, WA, USA), and the anti-β-tubulin (#E7) antibody from DSHB (Iowa City, IA, USA). Detection was carried out using HRP-conjugated secondary antibodies (anti-rabbit or anti-mouse IgG), followed by chemiluminescence visualization. Band intensities were quantified and normalized using β-tubulin as the loading control.

### 2.4. qRT-PCR Analysis

Total RNA was isolated from Caco-2 cells using TRIzol reagent (Sigma) and reverse transcribed with the iScript kit (Bio-Rad, Hercules, CA, USA). Quantitative PCR was then conducted using iQ SYBR Green Supermix (Bio-Rad) on the CFX384 RT-PCR detection system (Bio-Rad). To avoid genomic DNA amplification, primers spanning exon–exon junctions were designed (Supplementary Table S1), with 18S rRNA serving as the internal control.

### 2.5. Gas Chromatography–Mass Spectrometry (GC–MS) Analysis of Extracellular Lactate in Cell Culture Medium

For extracellular metabolite analysis, the cell culture media were collected and homogenized with two volumes of methanol containing ribitol (25 μg/mL) as an internal standard. Metabolite extracts were dried, derivatized, and analyzed by GC–MS following our previously described method [21]. Helium served as the carrier gas, with a front inlet purge flow of 3 mL/min and a column flow rate of 1 mL/min. The temperature program began at 50 °C (held for 2 min), followed by a ramp of 5 °C/min to 100 °C (held for 10 min), then 10 °C/min to 200 °C (held for 10 min), and finally 20 °C/min to 300 °C. Lactate abundances was determined using peak area ratios of analytes to the internal standard. Lactate standard was purchased from Sigma.

### 2.6. Wound Healing Assay

Cell migration was evaluated using a wound healing assay. Briefly, 1 × 10^6^ Caco-2 cells were plated in 12-well plates and allowed to reach confluence. A sterile 200 μL pipette tip was used to create a circle wound at the center of each well. Detached cells and debris were removed by gently washed with 1 × Phosphate-Buffered Saline (PBS), followed by imaging of the initial wound area. Cells were then cultured in DMEM containing 1% FBS, 100 units/mL penicillin G, and 100 μg/mL of streptomycin. The reduced serum concentration was used to suppress cell proliferation and focus on migration. Treatment with PP extract and hypoxia were applied as described in Section 2.2. After 48 h, wound areas were re-imaged, and extent of closure was quantified using ImageJ software (Version 1.53, National Institutes of Health, Bethesda, MD, USA). The percentage of wound closure was calculated using the formula:

Percentage of wound area=1−(Initial wound area−Final wound area)/(Initial wound area)

### 2.7. Cell Proliferation Assay

Cell proliferation was measured using the MTT (3-(4,5-Dimethylthiazol-2-yl)-2,5-Diphenyltetrazolium Bromide) assay. Caco-2 cells were diluted to 10^6^ cells/mL and seeded into 96-well plates (100 µL per well). After a 12-h incubation, cells were treated with 40 μg/mL PP extract and subjected to hypoxia, as previously described. Following 48 h of treatment, 10 µL of MTT solution (Sigma, 5 mg/mL in 1 × PBS) was added to each well and incubated for 4 h to allow formazan crystals formation. The medium was then removed, and 100 µL of dimethyl sulfoxide (DMSO, J.T.Baker, Center Valley, PA, USA) was added to solubilize the crystals. Absorbance at 570 nm was recorded using a BioTek Synergy H1 reader (Agilent Technologies, Palo Alto, CA, USA).

### 2.8. Drug Resistance Assay

Drug resistance was evaluated using the MTT assay, with minor modifications from the procedure described in Section 2.7. Caco-2 cells were seeded into 96-well plates (10^6^ cells/mL, 100 µL per well) and allowed to attach for 12 h. Cells were then treated with 40 μg/mL PP extract and 5-Fluorouracil (5FU, Sigma) at concentrations ranging from 0 to 10 µM [23,24]. Cells were then incubated under normoxic or hypoxic conditions for 48 h. All subsequent steps, including MTT reagent addition, incubation, formazan solubilization with DMSO, and absorbance measurement at 570 nm, were carried out as described in Section 2.7.

### 2.9. Statistical Analyses

Statistical analysis was performed using GraphPad Prism 7. Results are expressed as mean ± standard error of the mean (SEM). One-way ANOVA was used to assess significance, with *p*-value ≤ 0.05 considered significant.

## 3. Results

### 3.1. PP Extract Suppresses Glycolysis and Improves Oxidative Phosphorylation Under Hypoxia Stress

**Figure 1:**
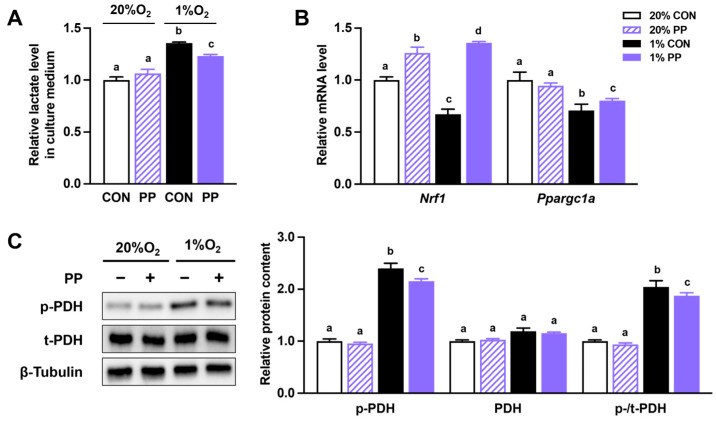
Purple potato extract suppresses glycolysis and improves oxidative phosphorylation under hypoxia. (**A**) Extracellular lactate content. (**B**) Relative mRNA level of peroxisome proliferator-activated receptor gamma coactivator 1 (*Ppargc1a*) and nuclear respiratory factor (*Nrf1*). (**C**) Total and phosphorylated pyruvate dehydrogenase (p-/t-PDH) protein levels. Cells were incubated under normoxia (20% O_2_) or hypoxia (1% O_2_). CON: cells without 40 μg/mL purple potato (PP) extract supplementation. PP: cells supplemented with 40 μg/mL PP extract. Data are shown as mean ± SEM (*n* = 3–4). Bars labeled with different letters represent statistically significant differences (*p* ≤ 0.05).

PDH phosphorylation was elevated under hypoxia, while PP extract supplementation mitigated this increase (Figure 1C). The lactate content in the culture media, serving as an indicator of glycolytic activity, showed a similar trend, increasing under hypoxia and decreasing with PP extract treatment (Figure 1A). Furthermore, hypoxia downregulated the expression of *Ppargc1a* and *Nrf1*, while PP extract supplementation alleviated this suppression (Figure 1B).

### 3.2. PP Extract Suppresses HIF-1α Accumulation and Downstream Gene Expression

**Figure 2:**
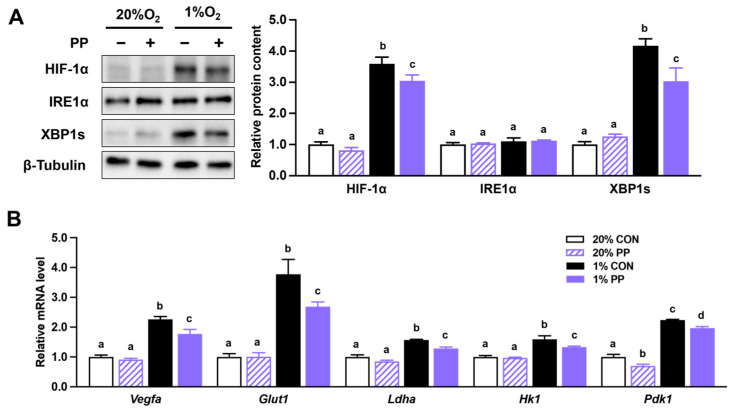
Purple potato extract inhibits HIF-1α and XBP1s accumulation and signaling in hypoxic cells. (**A**) Protein content of HIF-1α, inositol-requiring transmembrane kinase/endoribonuclease 1α (IRE1α), and XBP-1s. (**B**) Relative mRNA level of vascular endothelial growth factor a (*Vegfa*), glucose transporter 1 (*Glut1*), lactate dehydrogenase A (*Ldha*), hexokinase 1 (*Hk1*), and pyruvate dehydrogenase kinase 1 (*Pdk1*). Cells were incubated under normoxia (20% O_2_) or hypoxia (1% O_2_). CON: cells without 40 μg/mL purple potato (PP) extract supplementation. PP: cells supplemented with 40 μg/mL PP extract. Data are shown as mean ± SEM (*n* = 3). Bars labeled with different letters represent statistically significant differences (*p* ≤ 0.05).

Hypoxia enhanced HIF-1α protein accumulation in Caco-2 cells, which was reduced by PP extract treatment (Figure 2A). The spliced form of XBP1 was increased under hypoxic conditions and attenuated by PP extract supplementation (Figure 2A). Total IRE1α protein level remained unchanged (Figure 2A).

Additionally, the mRNA expression levels of HIF-1α target genes, including *Glut1*, *Ldha*, *Vegfa*, *Hk1*, and *Pdk1*, were elevated in response to hypoxia. These increases were attenuated by PP extract supplementation (Figure 2B).

### 3.3. PP Extract Suppresses Cell Proliferation and Migration Under Hypoxia Stress

**Figure 3:**
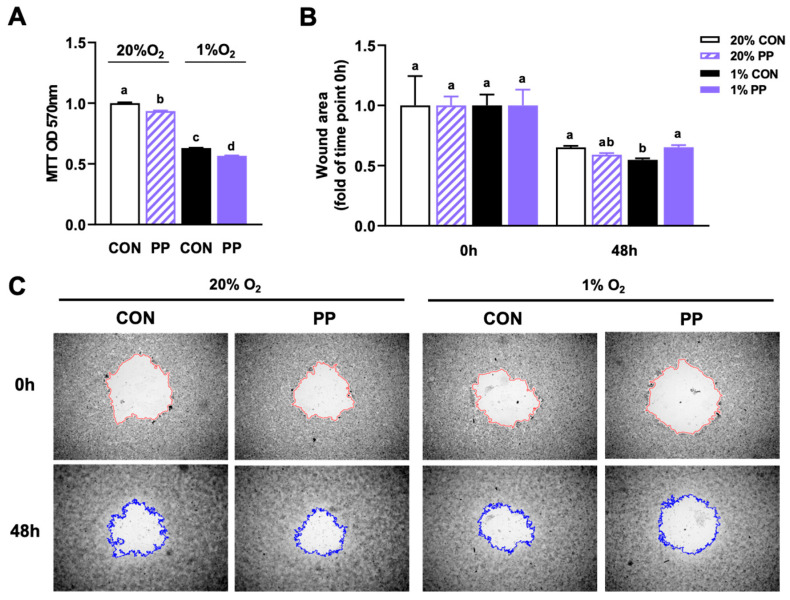
Purple potato extract suppresses cell proliferation and migration under hypoxia. (**A**) MTT assay of Caco-2 cell proliferation. (**B**) Quantification of cell migration based on wound area. (**C**) Representative wound images at 0 and 48 h post-wounding. Colored outlines indicate wound boundaries automatically generated using Image J. Cells were incubated under normoxia (20% O_2_) or hypoxia (1% O_2_). CON: cells without 40 μg/mL purple potato (PP) extract supplementation. PP: cells supplemented with 40 μg/mL PP extract. Data are shown as mean ± SEM (*n* = 3–6). Bars labeled with different letters represent statistically significant differences (*p* ≤ 0.05).

PP extract inhibited cell proliferation and migration under hypoxic conditions, as shown by the reduced cell viability and wound closure areas (Figure 3A–C). In addition, PP extract suppressed cell proliferation under normoxic conditions, indicating that its effects on cancer cell growth were not limited to hypoxic stress (Figure 3A).

### 3.4. PP Extract Suppresses Cell Drug Resistance and Stemness Gene Expression

**Figure 4:**
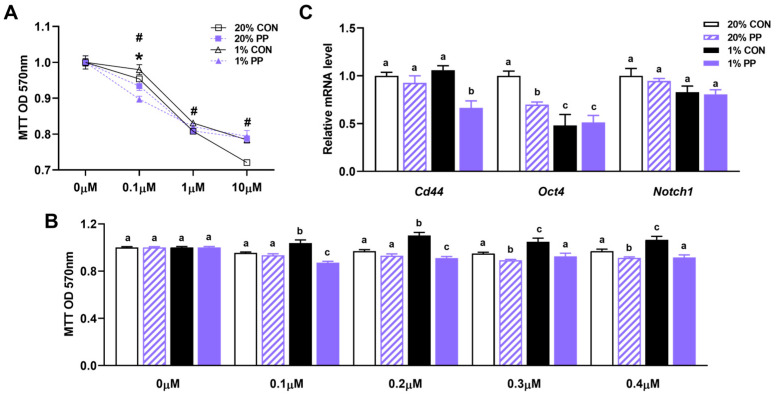
Purple potato extract reduces drug resistance of Caco-2 cells under hypoxia. (**A**) Dose-response of Caco-2 cell resistance to 5FU (0-10 µM). # indicates significant differences between the 20% CON and 1% CON groups, * indicates significant differences between the 1% CON and 1% PP groups. (**B**) Drug resistance of Caco-2 cells at low concentration of 5FU (0-0.4 µM), (**C**) Relative mRNA level of Cluster of differentiation 44 (*Cd44*), Notch homolog 1 (*Notch 1*), and Octamer-binding transcription factor 4 (*Oct4*). 20% CON: cells were incubated under normoxia (20% O_2_) without 40 μg/mL PP extract; 20% PP: cells were incubated under normoxia (20% O_2_) with 40 μg/mL PP extract; 1% CON: cells were incubated under hypoxia (1% O_2_) without 40 μg/mL PP extract; 1% PP: cells were incubated under hypoxia (1% O_2_) with 40 μg/mL PP extract. Data are shown as mean ± SEM (*n* = 3–5). Bars labeled with different letters represent statistically significant differences (*p* ≤ 0.05).

Caco-2 cells exhibited resistance to 5FU at concentrations ranging from 0.1 to 10 µM under hypoxic conditions, as indicated by sustained cell viability despite drug treatment (Figure 4A). Treatment with PP extract attenuated this resistance, with a notable increase in 5FU sensitivity observed particularly at concentrations between 0.1 to 0.4 μM (Figure 4B).

Analysis of stemness-related gene expression revealed that hypoxia had minimal impact on *Cd44* and *Notch1* levels, while *Oct4* expression reduced (Figure 4C). Under hypoxia conditions, PP extract decreased the expression of *Cd44*, and under normoxic conditions, it reduced the gene expression of *Oct4* (Figure 4C). *Notch1* expression remained largely unchanged across all conditions.

## 4. Discussion

This study reveals that PP extract mitigates the migration, proliferation, and drug resistance of colon cancer cells under hypoxia conditions by inhibiting glycolysis and HIF-1α signaling. Under hypoxia conditions, cells undergo metabolic adaptions to cope with the reduced oxygen availability, which promotes cancer progression by enhancing glycolysis gene transcription [25]. PDH, a key enzyme that regulates glycolysis and lactate production, is inactivated under hypoxia through phosphorylation by PDK [26]. The modification redirects pyruvate from mitochondrial oxidative phosphorylation toward lactate production [26]. Hypoxia also disrupts protein folding in the ER, triggering ER stress and activating the UPR [11]. URP contributes to cancer progression by stabilizing and activating HIF-1α, thereby inducing genes involved in cell proliferation, metabolic adaptation, and angiogenesis [14,27,28]. XBP1, a key marker of ER stress, can form a transcriptional complex with HIF-1α to modulate the expression of its downstream target genes [15].

In line with known mechanisms, we observed increased phosphorylation of PHD and lactate production under hypoxia, both of which were effectively reversed by PP extract, indicating its potential to restore metabolic balance under oxygen-deprived conditions. These transcriptional changes indicate that PP extract interferes with hypoxia-driven gene programs critical to tumor adaptation. Moreover, PP extract reduced XBP1 and HIF-1α levels, as well as the expression of HIF-1α target genes associated with energy production (*PDH*, *Pdk1*, *HK1*, *Glut1*) and angiogenesis (*Vegfa*) under hypoxia. These results indicate that PP extract may suppress HIF-1α signal and ER stress-associated metabolic adaptation under hypoxia. Importantly, it remains to be determined whether the inhibitory effects of PP extract on HIF-1α and XBP1 result from transcriptional downregulation, post-translational modifications, or disruption of upstream signaling pathways. Similar to our findings, natural compounds such as alkannin, apigenin, and catechin can inhibit HIF signaling and glycolysis-related factors, thereby impeding cancer progression [19,20,29].

Our previous work showed that PP extract promotes mitochondrial function and oxidative phosphorylation in Caco-2 cells [21]. In the current study, we further found that it alleviates hypoxia-induced suppression of *Ppargc1a* and *Nrf1* expression. NRF1 is essential for mitochondrial biogenesis and oxidative phosphorylation [30], while PGC-1α is a key transcriptional coactivator that regulates mitochondrial energy metabolism, partly by activating NRF1 [30,31]. HIF-induced transcriptional changes under hypoxia typically favor glycolysis over oxidative metabolism, thereby suppressing the activity of PGC-1α and NRF1 [32,33]. By restoring their expression, PP extract may help maintain mitochondrial respiratory capacity, reduce reliance on anaerobic glycolysis, and promote a more oxidative metabolic phenotype in cancer cells. Our findings suggest that PP extract may counteract this metabolic reprogramming and support mitochondrial function even under hypoxic stress.

Additionally, the ability of PP extract to limit hypoxia-driven proliferation and migration highlights its potential to counteract metastatic progression in CRC. Since chemoresistance is often correlated with enhanced glycolysis and increased expression of stemness-related genes [34], we further investigated the impact of PP extract on 5FU resistance and stemness gene expression. 5FU, a widely used chemotherapy drug for CRC, targets thymidylate synthase, disrupting DNA synthesis and causing death in rapidly dividing cancer cells [35]. However, hypoxia also contributes to drug resistance by altering drug metabolism, activating survival pathways, enhancing DNA repair, and suppressing apoptosis [36]. A key mediator of these effects is the HIF pathway, whose activation promotes glycolysis and lactate accumulation, creating an acidic extracellular environment that impairs drug penetration [37,38]. Consistently, in this study, hypoxia increased resistance to 5FU in Caco-2 cells at concentrations ranging from 0.1 μM to 0.4 μM. Notably, co-treatment with PP extract attenuated this resistance, potentially by reducing lactate production and thus alleviating extracellular acidification, which may improve drug delivery to cancer cells [39,40]. The observation is particularly relevant given that hypoxic tumor regions are known to respond poorly to chemotherapy in clinical settings. Thus, PP extract may provide an adjuvant strategy to enhance drug delivery and restore chemosensitivity. While we did not formally assess synergism using combination index analysis or dose–response matrices, our findings suggest that PP extract may enhance 5FU efficacy under hypoxia. Further studies are warranted to determine whether this effect is synergistic, which would have important implications for improving chemotherapeutic outcomes in hypoxic tumors.

While hypoxia often activates the HIF pathway and enhances the stemness gene expression [41,42], the response can be gene- and context-specific. For instance, some studies have reported that hypoxia reduces the expression of certain stemness genes, while in others, it promotes their expression [43,44,45]. Consistent with findings in embryonic stem cells and primordial germ cells [43,44], we observed that hypoxia reduced *Oct4* expression. Regarding the effect of PP extract, our results indicate a limited and gene-specific modulation of stemness markers: it downregulated *Cd44* under hypoxia and *Oct4* under normoxia, while *Notch 1* expression remained largely unchanged. These differential effects suggest that PP extract may not uniformly target stemness pathways but instead acts selectively depending on environmental conditions and gene context. It is also possible that the limited response observed is due to the short exposure time or that PP extract primarily influences metabolic rather than stemness-related pathways. Further studies with extended treatment durations and broader profiling are needed to clarify its effects on cancer stem cell characteristics.

While our findings provide promising evidence that PP extract modulates hypoxia-associated metabolic pathways and may enhance chemotherapeutic efficacy, this study remains preliminary. Further comprehensive investigations using larger sample sizes, in vivo models, and mechanistic analysis are needed to validate these effects and elucidate the underlying molecular mechanisms.

## 5. Conclusions

PP extract mitigates hypoxia-induced metabolic reprogramming, cell proliferation, migration, and drug resistance in Caco-2 cells by inhibiting HIF-1α signaling and suppressing glycolysis. Additionally, PP extract enhances oxidative phosphorylation and downregulates the select stemness genes. Future investigations are needed to elucidate the underlying molecular mechanisms and to conduct *in vivo* studies that further elucidate the therapeutic potential of PP extract in cancer treatment. These findings underscore the potential of PP extract as a natural dietary intervention to counteract hypoxia-driven cancer progression and enhance the efficacy of chemotherapy in CRC.

## Data Availability

Data is contained within the article or Supplementary Material.

## Supplementary Materials

The following supporting information can be downloaded at: https://www.mdpi.com/article/10.3390/nu17132079/s1, Table S1: Lists the primer sets used for quantitative RT-qPCR.
