# Supplementary material for: Purple Potato Extract Suppresses Hypoxia-Induced Metabolic Reprogramming and Inhibits HIF-1α Signaling in Caco-2 Cells
Source: Nutrients. 2025 Jun 23;17(13):2079. doi: 10.3390/nu17132079 (PMC12251104; doi:10.3390/nu17132079)
Supplement: Supplementary file 1 [file nutrients-17-02079-s001.zip › nutrients-3681329-supplementary.pdf]

Supporting Information for Online Publication

Supplementary Table 1. Primer sequences used for RT-qPCR analyses

Gene name	Accession No.	Product size (bp)	Sequence (5'→3')
18s	NR_146119.1	76	F: CGCACGGCCGGTACAGTGAA
			R: GGGAGAGGAGCGAGCGACCA
Vegfa	NM_001025366.3	71	F: GGCCAGCACATAGGAGAGAT
			R: GCTCTATCTTTCTTTGGTCTGCA
Glut1	NM_006516.4	133	F: TTTGGCCGGCGGAATTCAAT
			R: CGCAGTACACACCGATGATG
Ldha	NM_001135239.2	178	F: GACGTGCATTCCCGATTCC
			R: TCATCTGCCAAGTCCTTCATTA
Hk1	NM_000188.3	88	F: AAGATCCGCGAGAACAGAGG
			R: TGGAGAAGTGTGGATGAAGCT
Pdk1	NM_001278549.2	98	F: GTCACCAGCCAGAATGTTCA
			R: CCACCAAACAATAAAGAGTGCTG
Nrf1	NM_001040110	102	F: AGGAACACGGAGTGACCCAA
			R: TATGCTCGGTGTAAGTAGCCA
Ppargc1a	NM_001330751.2	112	F: TCTGAGTCTGTATGGAGTGACAT
			R: CCAAGTCGTTCACATCTAGTTCA
Oct4	NM_001173531.3	164	F: CTTGAATCCCGAATGGAAAGGG
			R: GTGTATATCCCAGGGTGATCCTC
Notch1	NM_017617	140	F: GAGGCGTGGCAGACTATGC
			R: CTTGTACTCCGTCAGCGTGA
Cd44	NM_001001392	109	F: CTGCCGCTTTGCAGGTGTA
			R: CATTGTGGGCAAGGTGCTATT
